# Supplementary material for: Robust total X-ray scattering workflow to study correlated motion of proteins in crystals
Source: Nat Commun. 2023 Mar 3;14:1228. doi: 10.1038/s41467-023-36734-3 (PMC9984388; doi:10.1038/s41467-023-36734-3)
Supplement: Supplementary file 1 — Supplementary Information [file 41467_2023_36734_MOESM1_ESM.pdf]

Supplementary Information for: Robust total X-ray scattering workflow to study correlated motion of proteins in crystals

Steve P. Meisburger[1] David A. Case[2] Nozomi Ando[1*]

[1] Department of Chemistry and Chemical Biology, Cornell University, Ithaca, New York 14850, USA.

[2] Department of Chemistry and Chemical Biology, Rutgers University, Piscataway, New Jersey 08854, USA.

* Correspondence to: nozomi.ando@cornell.edu

Supplementary Table 1. Data collection parameters

Space group	Crystal	Sweep	Frames	Rotation (deg.)	Image file prefix
$P2_12_12_1$	1	1	1–500	0–50	lys_rt_2_38
		2	1–500	90–140	lys_rt_2_39
		3	1–500	180–230	lys_rt_2_40
		4	1–500	270–320	lys_rt_2_41
	2	1	1–500	0–50	lys_rt_5_51
		2	1–500	90–140	lys_rt_5_52
		3	1–500	180–230	lys_rt_5_53
		4	1–500	270–320	lys_rt_5_54
$P3_32_12$	1	1	1–500	247–297	lys_1_2
		2	1–500	292–342	lys_1_3
		3	1–500	237–387	lys_1_4
		4	1–500	22–72	lys_1_5
		5	1–500	67–117	lys_1_6
		6	1–500	112–162	lys_1_7
		7	1–500	157–207	lys_1_8
		8	1–500	202–252	lys_1_9

Supplementary Table 2. Bragg data collection and structure refinement statistics. *Values in parentheses refer to the highest resolution shell.

	PDB ID: *8dz7*	PDB ID: *8dyz*
**Data Collection**		
Space Group	P $2_1 2_1 2_1$	P $4_3 2_1 2$
Cell dimensions		
a, b, c (Å)	30.49, 56.40, 73.85	79.63, 79.63, 38.30
α, β, γ (°)	90, 90, 90	90, 90, 90
Resolution (Å)	44.87–1.34 (1.36–1.34)*	39.85–1.27 (1.29–1.27)
R_{merge}	0.039 (0.238)	0.046 (0.380)
R_{pim}	0.011 (0.170)	0.009 (0.184)
I / σI	36.0 (3.8)	39.2 (3.7)
$CC_{1/2}$	0.999 (0.898)	1.000 (0.878)
Completeness (%)	96.9 (68.4)	98.0 (78.1)
Multiplicity	12.1 (2.2)	23.8 (4.6)
**Refinement**		
Resolution (Å)	44.87–1.34	39.85–1.27
Unique reflections: all/free	28632 / 1417	32215 / 1659
R_{work} / R_{free}	0.118 / 0.136	0.115 / 0.133
Number of non-H atoms		
Protein	1026	1030
Ligand/ion	2	4
Water	50	78
Mean isotropic B-factors		
Protein	24.80	25.45
Ligand/ion	24.55	40.2
Water	34.32	35.76
Model validation		
Ramachandran outliers (%)	0.00	0.00
Ramachandran favored (%)	99.21	99.21
Rotamer outliers (%)	0.00	0.00
C-β deviations	0	0
R.m.s. bond lengths (Å)	0.0175	0.0160
R.m.s. bond angles (°)	1.97	1.85
Clashscore	0.00	0.00
Overall score	0.5	0.5

Supplementary Table 3. Diffuse data processing and lattice disorder model statistics

	Triclinic	Orthorhombic	Tetragonal
	(P 1)	(P $2_12_12_1$)	(P 4_32_12)
**Diffuse Map**			
Laue Group	*-1*	*mmm*	*4/mmm*
Reciprocal cell dimensions			
a*, b*, c* ($Å^{-1}$)	0.0416, 0.0337, 0.0307	0.0328, 0.0177, 0.0135	0.0126, 0.0126, 0.0261
α, β, γ (°)	83.82, 70.60, 67.35	90, 90, 90	90, 90, 90
Resolution (Å)	25–1.25	20–1.28	33–1.16
Voxel size (a*, b*, c*)	1/13, 1/11, 1/11	1/13, 1/7, 1/5	1/5, 1/5, 1/11
Merged observations ($\times 10^6$)	43.9	13.2	9.9
Completeness (%)	97.8	92.0	89.0
**DISCOBALL**			
3D-ΔPDF			
Resolution cutoff (Å)	1.25	1.62	1.62
Voxel size (a, b, c)	1/45, 1/54, 1/56	1/40, 1/70, 1/96	1/100, 1/100, 1/48
Deconvolution			
Number of peaks in the asu	787	84	36
Radius cutoff (Å)	4.0	4.0	4.0
Resolution range (Å)	5–1.67	5–1.67	5–1.67
**GOODVIBES**			
Network model			
Supercell (a, b, c)	13, 11, 11	13, 7, 5	5, 5, 11
Rigid groups (per unit cell)	1	4	8
Contacts (per asu)	310	232	202
Unique Contacts	155	116	102
Reference halos			
Resolution range (Å)	2.5–2.0	2.5–2.0	2.5–2.0
Number of halos	400	400	400
Number of voxels	621,858	166,773	101,265
Simulation			
Resolution range (Å)	∞–1.25	∞–1.62	∞–1.62
Atomic displacement parameters			
Protein center of mass (Å)	-1.01, 14.46, 24.33	-2.84, 12.51, -15.84	-0.59, 20.68, 19.41
Center of reaction (Å)	-1.85, 13.09, 25.77	-3.15, 12.70, -12.80	2.33, 22.82, 21.15
$T_{1,1}$, $T_{2,2}$, $T_{3,3}$ ($Å^2$)	0.0362, 0.0450, 0.0350	0.1091, 0.0855, 0.1126	0.0942, 0.1138, 0.1278
$T_{1,2}$, $T_{1,3}$, $T_{2,3}$ ($Å^2$)	-0.0003, 0.0040, 0.0022	-0.0120, 0.0023, -0.0085	0.0041, 0.0124, -0.0100
$L_{1,1}$, $L_{2,2}$, $L_{3,3}$ (deg.^{2})	0.5116, 0.5487, 0.5797	1.1164, 1.2407, 1.4753	1.7048, 1.3492, 1.1318
$L_{1,2}$, $L_{1,3}$, $L_{2,3}$ (deg.^{2})	-0.0196, -0.0566, -0.1044	-0.1860, 0.2218, -0.0708	-0.5496, 0.3593, -0.0113
$S_{1,1}$, $S_{2,2}$, $S_{3,3}$ (deg. Å)	0.0070, 0.0029, -0.0078	-0.0426, 0.0255, 0.0129	0.0244, -0.0034, -0.0149
$S_{1,2}$, $S_{1,3}$, $S_{2,3}$ (deg. Å)	-0.0061, 0.0034, -0.0004	0.0267, 0.0369, 0.0076	-0.0184, 0.0015, 0.0383

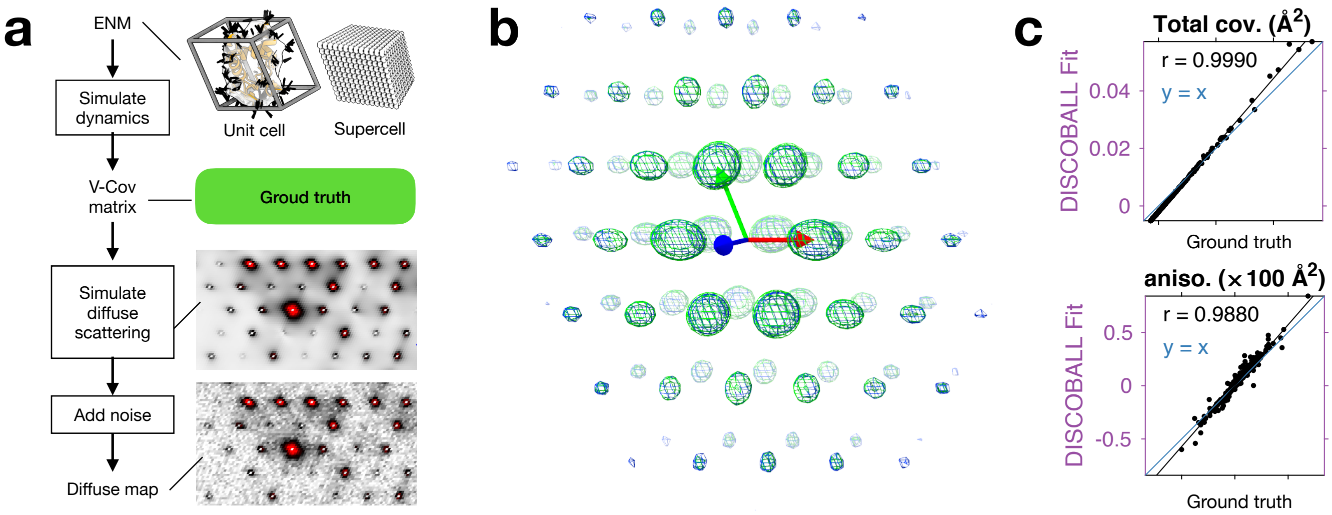

Supplementary Fig. 1. Validation of GOODVIBES models using DISCOBALL. **a**, A synthetic diffuse scattering dataset of lysozyme in space group P1 was generated using a GOODVIBES ENM model with random spring constants. The ground truth joint-ADPs were calculated from the variance-covariance matrix (V-Cov). Uniform Gaussian random noise was added to the map, and DISCOBALL was applied to estimate the joint-ADPs. **b**, Overlay of the true (green) and DISCOBALL (blue) joint-ADPs represented using isosurface ellipsoids. **c**, Quantitation of DISCOBALL's ability to estimate joint-ADP ellipsoids. DISCOBALL recovers both the overall displacement covariance (top panel) and the anisotropic components (bottom panel) with high correlation (r, Pearson correlation coefficient).

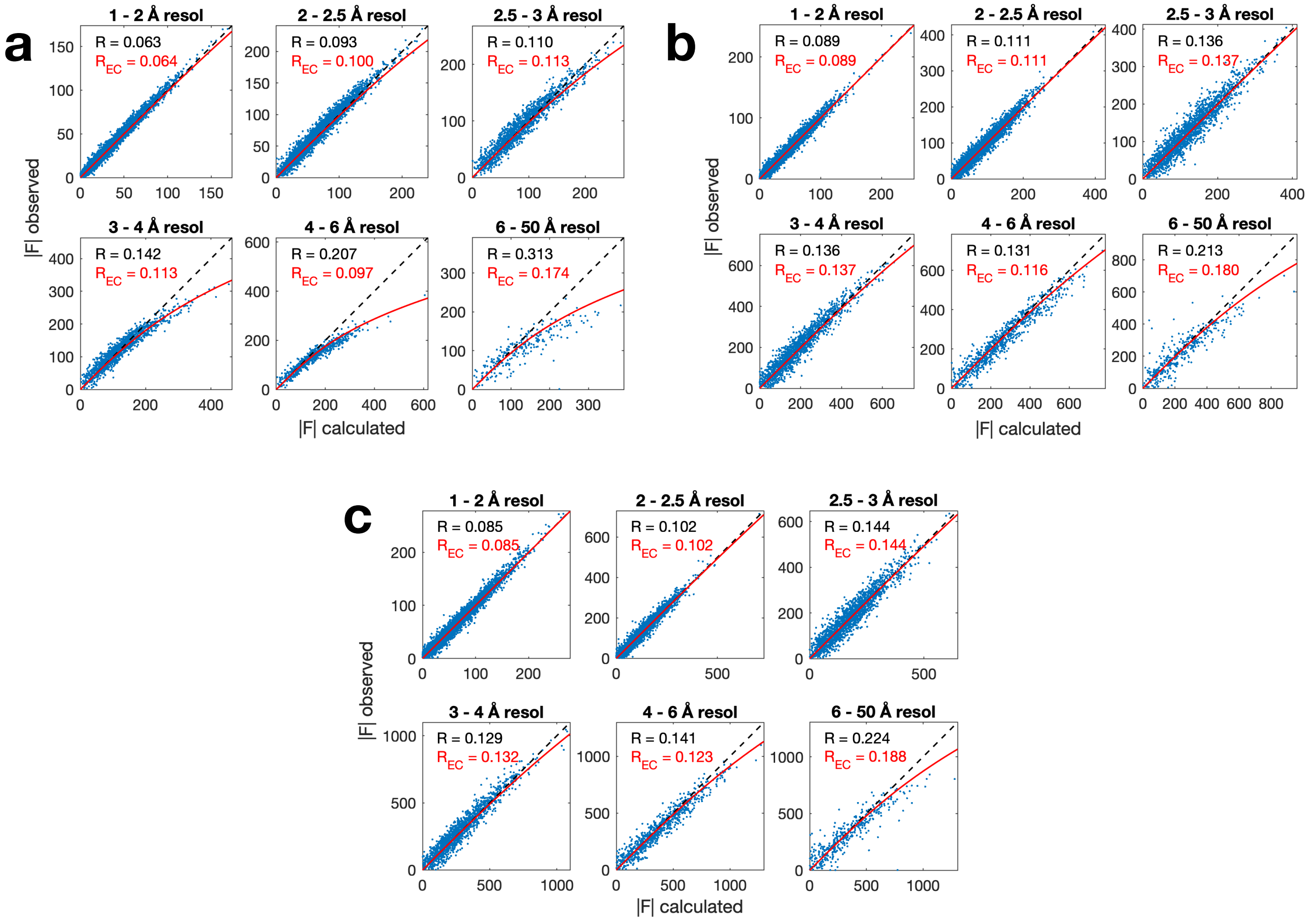

Supplementary Fig. 2. Apparent extinction of Bragg intensities from lysozyme polymorphs. Accurate structure factor amplitudes are needed for GOODVIBES simulations of diffuse scattering. We therefore investigated the source of discrepancies between observed structure factors (Fobs) and those calculated from the refined structure (Fcalc). **a**, Scatter plots of Fobs vs. Fcalc for triclinic lysozyme grouped by resolution (indicated above each panel). Especially at low resolution (bottom row), Fobs is systematically lower than Fcalc in an intensity-dependent manner (points fall below the dashed line in each panel). This discrepancy leads to high R-factors at low resolution (insets). The discrepancy fits the functional form of the extinction correction (EC) used in small molecule crystallography[1] when its free parameter x is optimized (red line, $x = 0.002$). After applying the EC, R-factors drop dramatically (R_{EC}, inset). **b**, Discrepancies between Fobs and Fcalc are also significant in orthorhombic lysozyme, but less so than in triclinic. Modest improvements in R-factor are seen when EC is applied (x=0.0001). **c**, Tetragonal lysozyme also shows slight discrepancies between Fobs and Fcalc at low resolution, and EC (x=0.00005) results in lower R-factors. Detector count-rate artifacts[2] might also explain the suppressed Bragg intensities, and cannot be ruled out without further experiments.

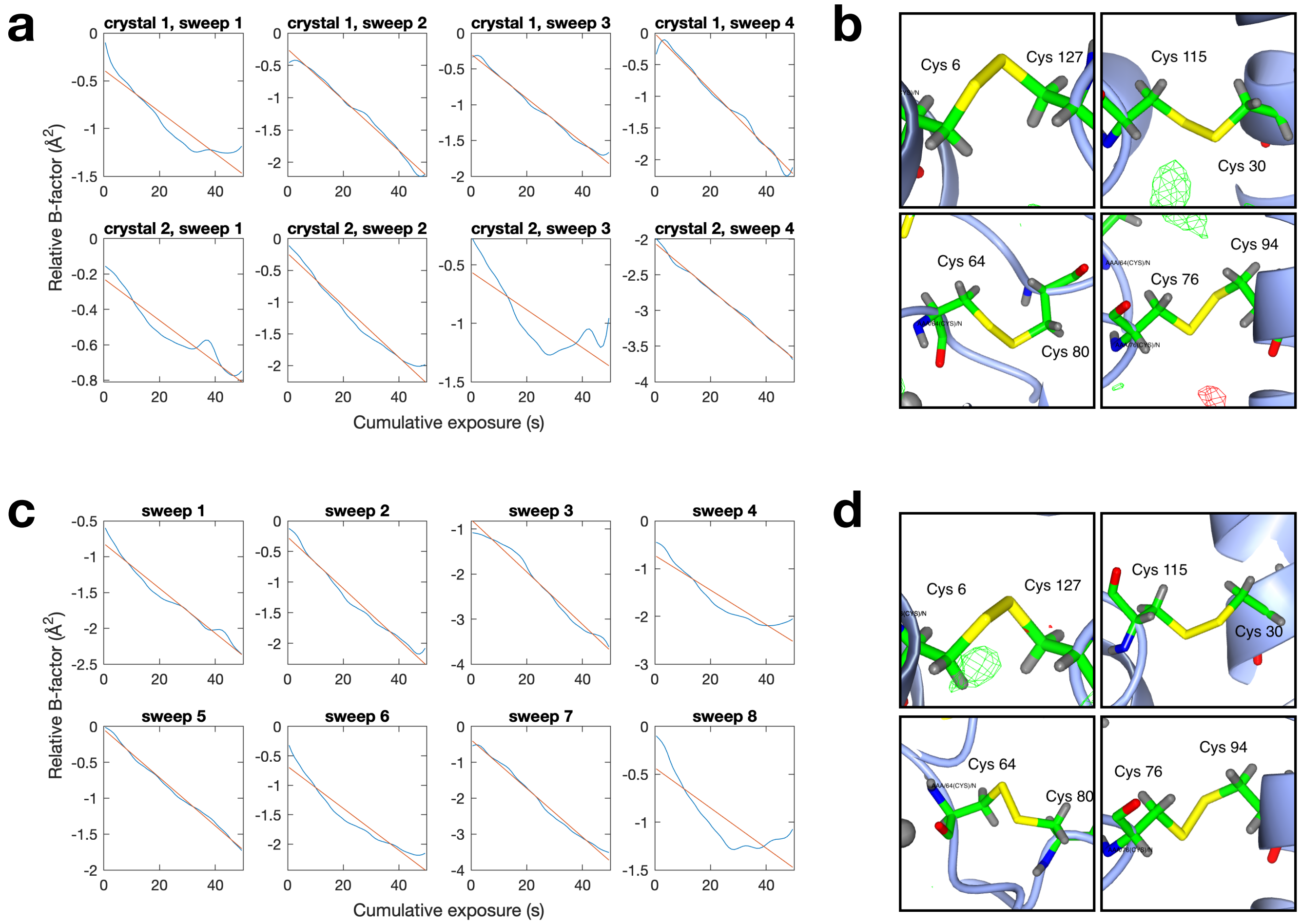

Supplementary Fig. 3. Monitoring global and site-specific radiation damage. Global damage was assessed using B-factor correction applied during scaling, and site-specific damage was assayed using the difference electron density around disulfide bonds. **a**, B-factor decay profiles of orthorhombic lysozyme reported by *Aimless*[3] (blue lines) show a characteristic linear decay with X-ray exposure (red fits). **b**, Difference density (fo-fc) is not observed near the four disulfide bonds of orthorhombic lysozyme at the 3σ level (green and red mesh) signifying that all four disulfides remained intact. **c**, B-factor decays for the tetragonal dataset are similar to orthorhombic (panel a). **d**, Little difference density is observed near the disulfide bonds of tetragonal lysozyme, except for a small positive feature near Cys 6 may signify a minor population with reduced sulfhydryl groups (not modeled).

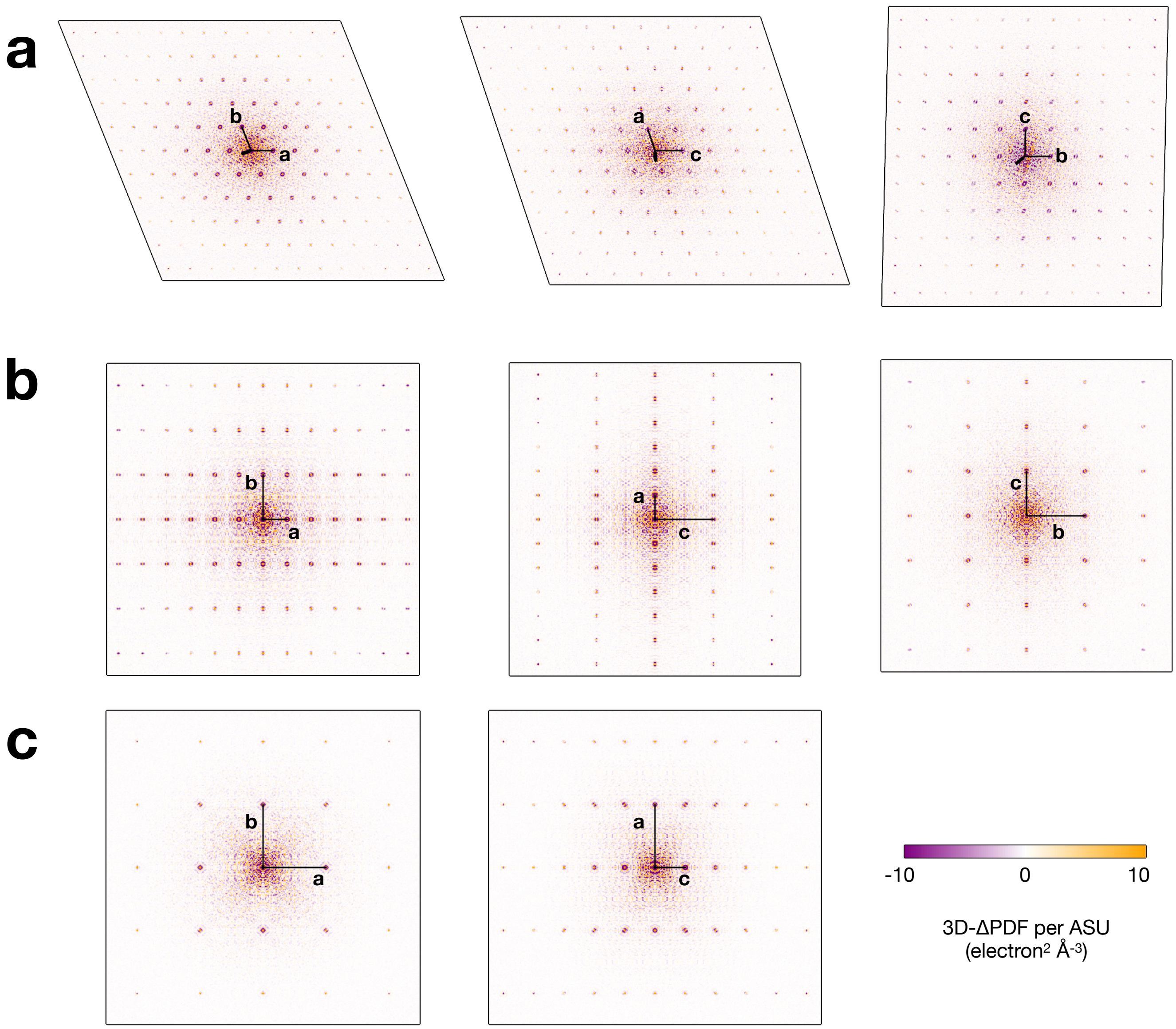

Supplementary Fig. 4. 3D-ΔPDFs of lysozyme polymorphs. The 3D-ΔPDF (Fourier transform of the diffuse scattering) was calculated for three experimental datasets from lysozyme as described in Methods. Central sections through the 3D-ΔPDF for crystals in the triclinic (panel **a**), orthorhombic (panel **b**), and tetragonal (panel **c**) space groups show intense features near the origin and a series of sharp peaks that decay in intensity away from the origin. The sharp peaks occur at nodes of the direct lattice (multiples of the unit cell vectors **a**, **b**, and **c**). All maps are shown on the same scales of relative distance and intensity per asymmetric unit (ASU).

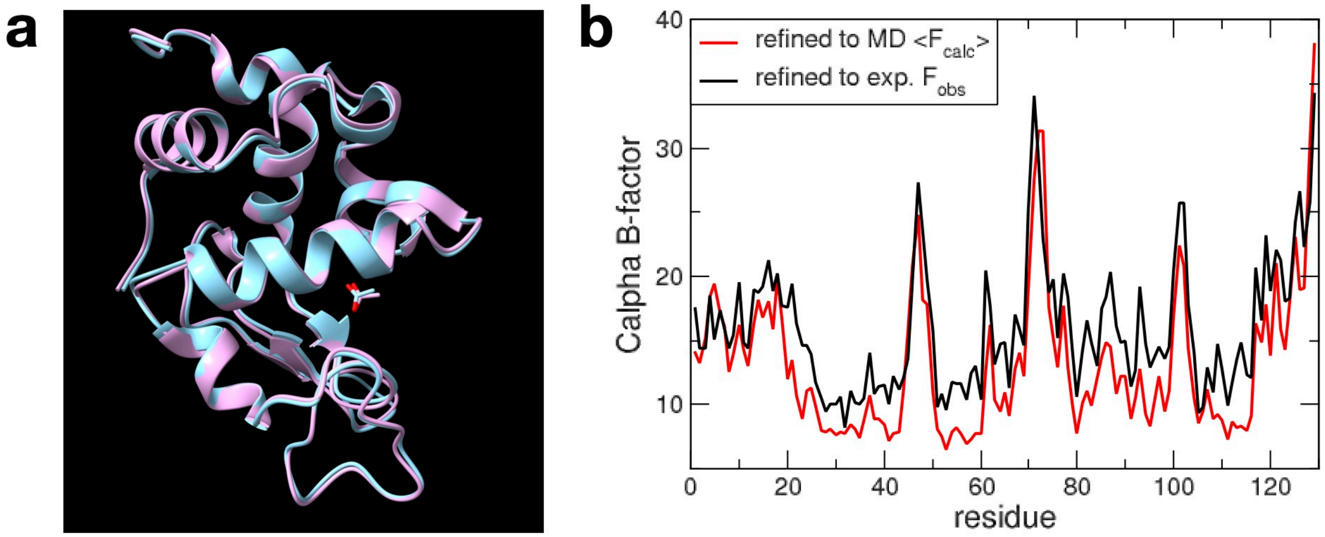

Supplementary Fig. 5. Comparison of MD simulation and experimental structures of lysozyme in tetragonal space group. **a**, The deposited structure is cyan, refined structure using the MD average structure factors is pink. **b**, Comparison between the refined B-factors for Cα atoms. The pattern is similar, although values refined against the MD data are somewhat lower in regions of secondary structure than are the values refined from experiment. Overall, these comparisons are very similar to those reported earlier for similar simulations of triclinic lysozyme[4].

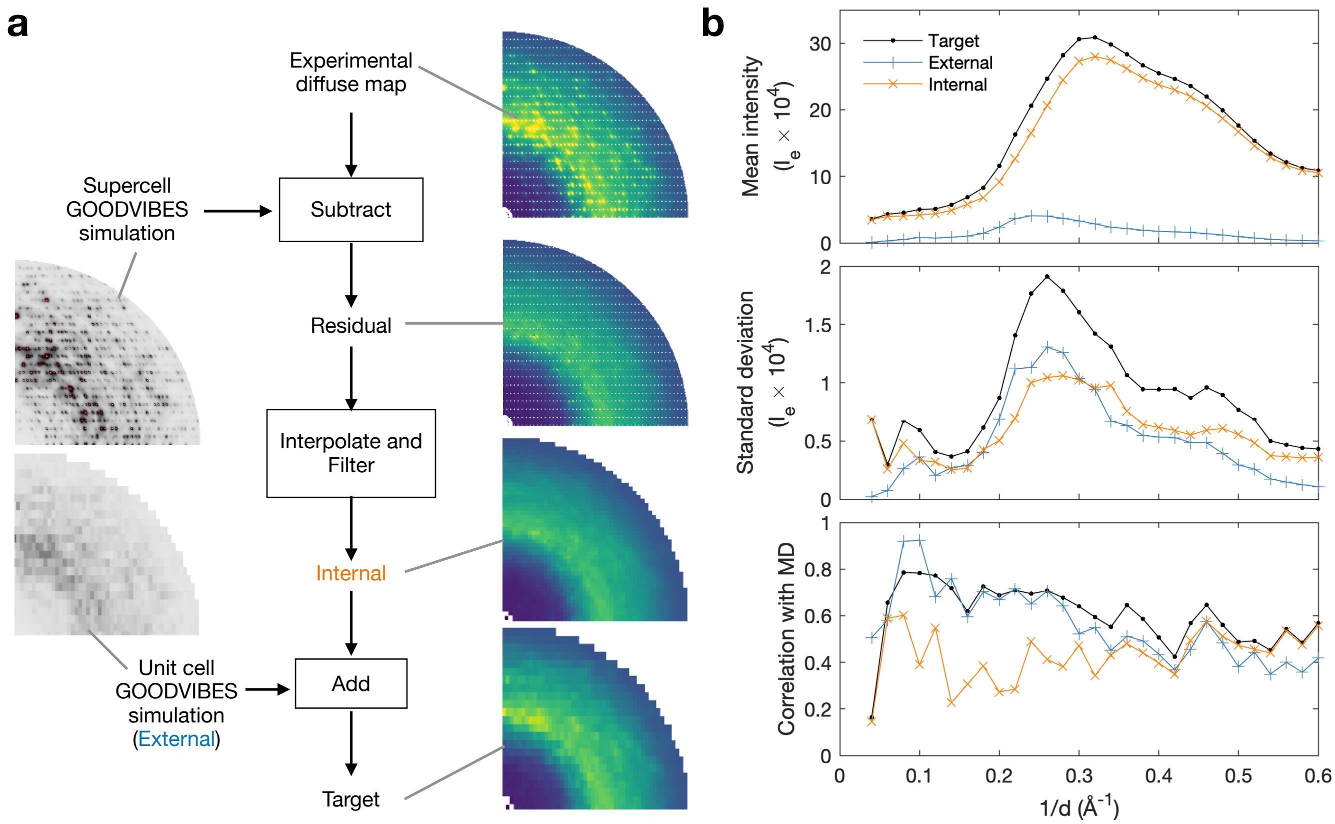

Supplementary Fig. 6. Components of the target diffuse map. a, Procedure for producing target diffuse maps illustrated using tetragonal lysozyme from an experimental diffuse map (top right panel). First a GOODVIBES simulation of lattice dynamics (left panel) is subtracted, and then a filter is applied in order to interpolate this residual signal onto an appropriate grid and remove outliers. External motions compatible with the MD simulation are simulated using GOODVIBES. Finally, the target diffuse map is computed as the sum of internal and external maps. See Methods in the Main Text for details. **b**, Contribution of signals from internal (orange lines and symbols) and external (blue lines and symbols) motions to the target map (black lines and symbols) for tetragonal lysozyme. Internal motion makes the main contribution to the isotropic signal (top panel), and the internal and external have a comparable contribution to the scattering variations (middle panel). The correlation coefficient with MD is dominated by the external signal at low resolution but internal motion becomes important at high resolution (bottom panel).

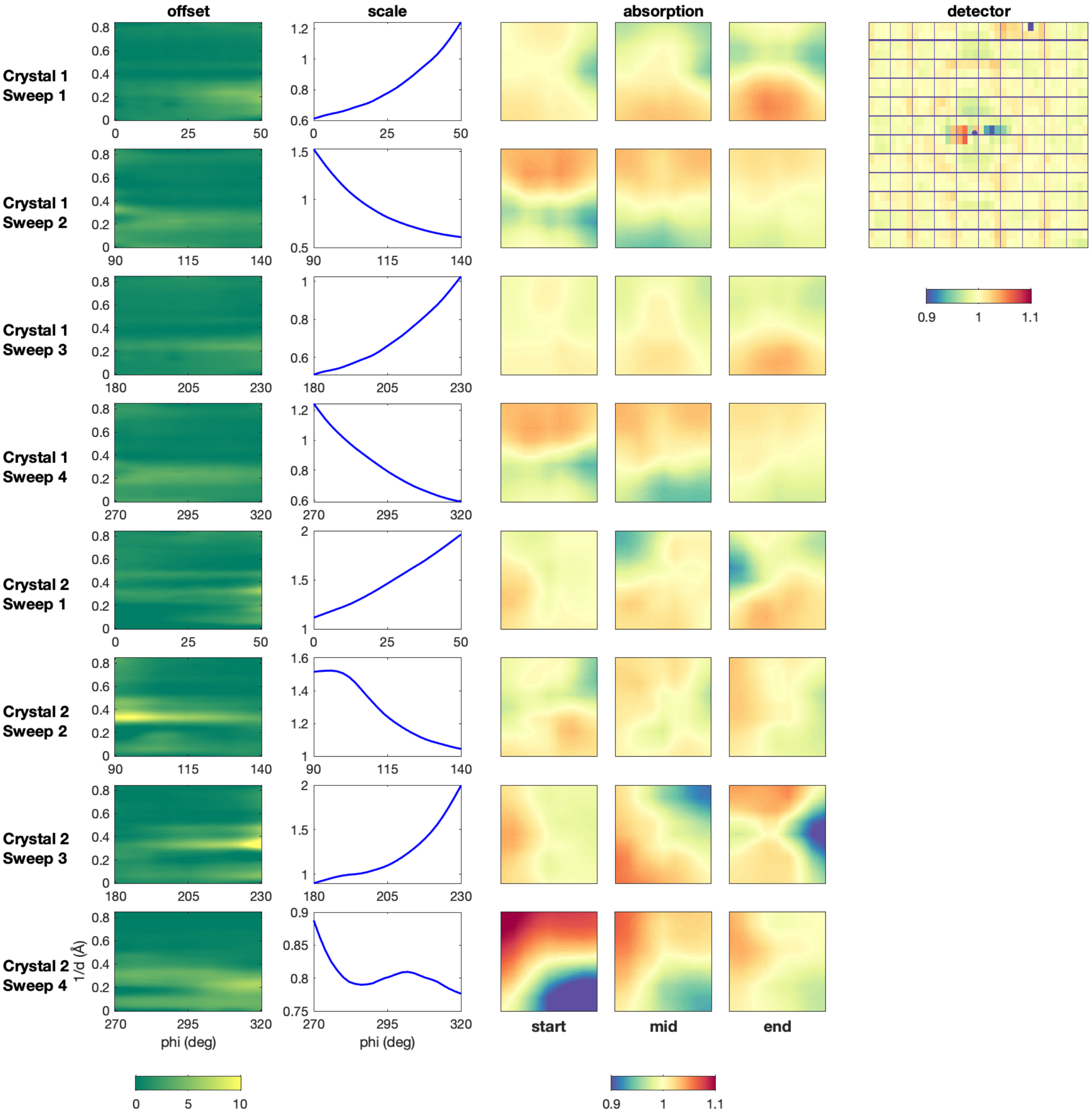

Supplementary Fig. 7. Refined scaling model to produce the diffuse map of orthorhombic lysozyme. A scaling model was refined to correct for experimental artifacts in X-ray images. The model relates the merged intensity to the observed intensity as a function of spindle angle, resolution (d), and detector position. From left to right, the model parameters included: (1) offset correction vs. spindle angle and resolution, (2) overall scale factor vs. spindle angle, (3) scale factor for absorption correction vs. spindle angle and detector position, and (4) scale factor for detector efficiency correction vs. detector chip index. The parameters in the model were refined in order to minimize the least-squares error between observation and prediction. The best-fit parameters are illustrated for each partial dataset (top to bottom) (refer to Supplementary Table 1).

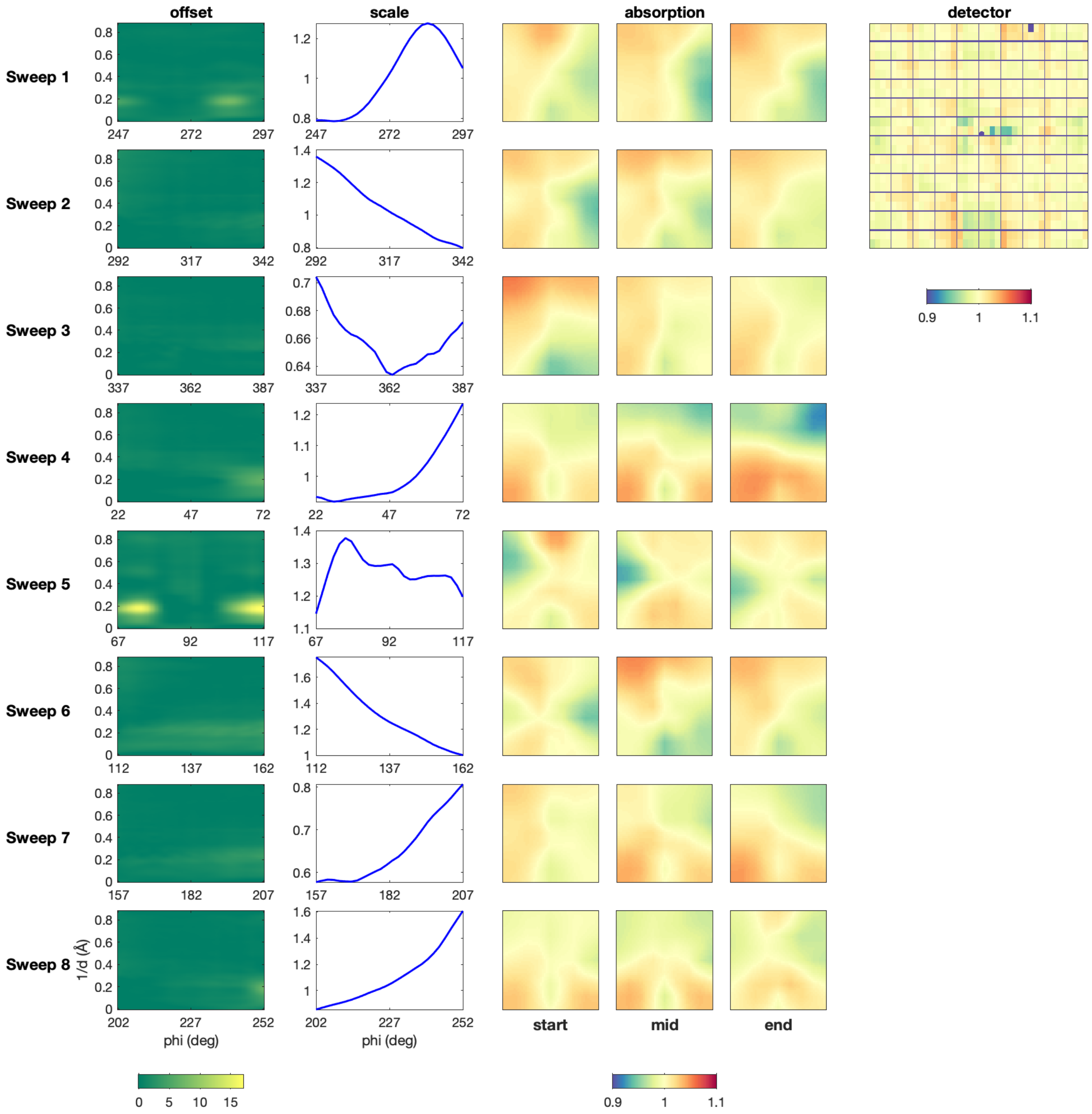

Supplementary Fig. 8. Refined scaling model to produce the diffuse map of tetragonal lysozyme. For explanation, see Supplementary Fig. 7.

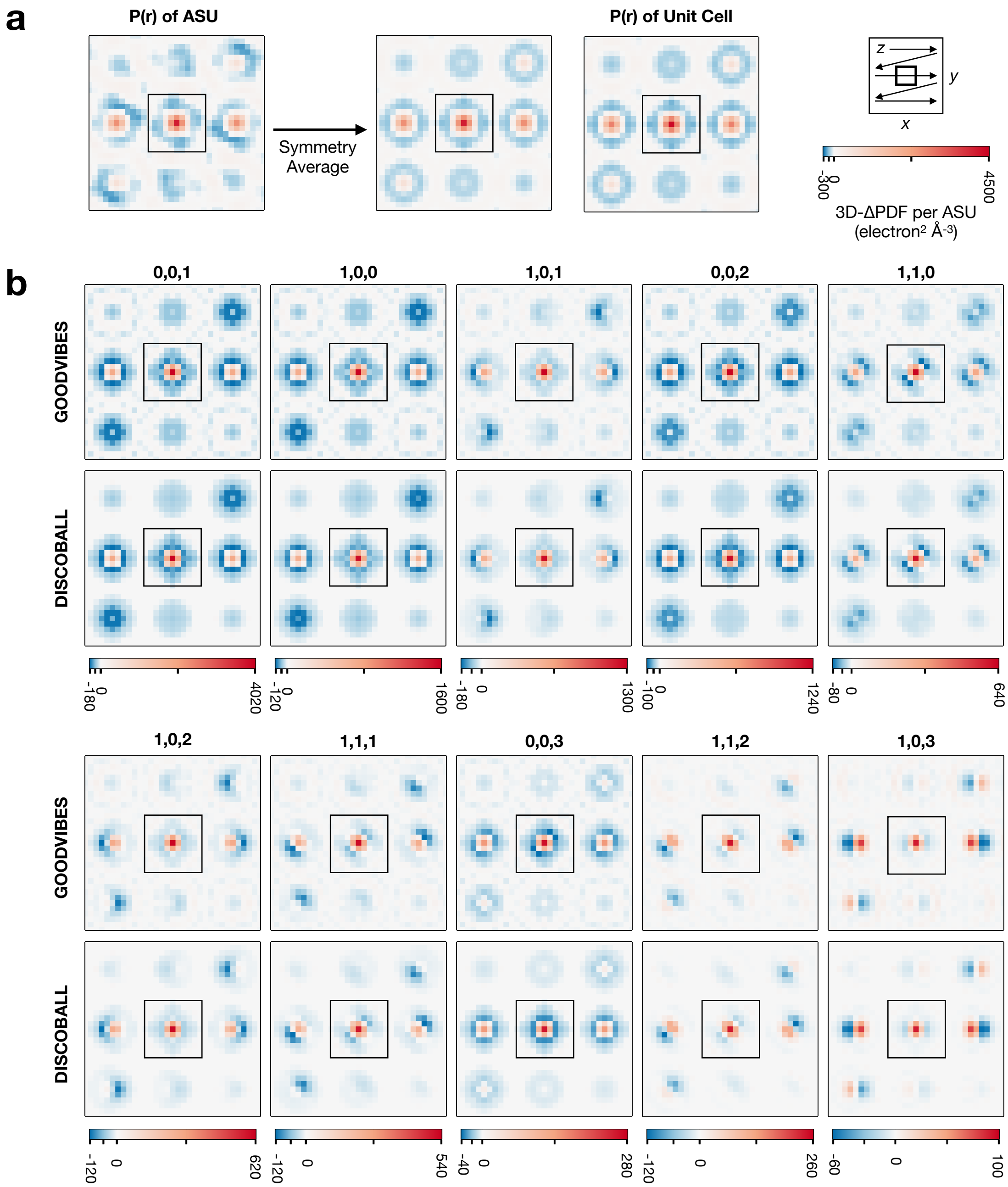

Supplementary Fig. 9. Test of approximations made in DISCOBALL model. The DISCOBALL model for 3D-ΔPDF peaks is approximate for symmetric space groups (see Methods in the Main Text). Using tetragonal lysozyme as an example, we tested the effect of these approximations compared with an exact simulation. **a**, The central peak in the autocorrelation (P(r)) of the asymmetric unit (ASU) is approximately invariant to symmetry transformations (compare the red density in the left and central panels). Furthermore, the peak in the symmetry averaged asymmetric P(r) is nearly identical to that of the conventional Patterson function (P(r) of the unit cell, right panel), confirming that cross-terms between asymmetric units can be neglected. **b**, The 10 most intense 3D-ΔPDF peaks were simulated exactly using GOODVIBES and approximated using the DISCOBALL model with effective joint-ADPs computed from the GOODVIBES model (no deconvolution was performed). The similarity between the two confirms that approximations used in DISCOBALL have minimal effect on predicting the peak shape in this case.

Supplementary References

1. Bourhis, L. J., Dolomanov, O. V., Gildea, R. J., Howard, J. A. & Puschmann, H. The anatomy of a comprehensive constrained, restrained refinement program for the modern computing environment–Olex2 dissected. *Acta Crystallogr., Sect. A: Found. Adv.* **71,** 59–75 (2015).
2. Trueb, P. *et al.* Bunch mode specific rate corrections for PILATUS3 detectors. *J. Synchrotron Radiat.* **22,** 701–707 (2015).
3. Evans, P. R. & Murshudov, G. N. How good are my data and what is the resolution? *Acta Crystallogr., Sect. D: Biol. Crystallogr.* **69,** 1204–1214 (July 2013).
4. Janowski, P. A., Liu, C., Deckman, J. & Case, D. A. Molecular dynamics simulation of triclinic lysozyme in a crystal lattice. *Protein Sci.* **25,** 87–102 (Jan. 2016).
