# Supplementary material for: Robust total X-ray scattering workflow to study correlated motion of proteins in crystals
Source: Nat Commun. 2023 Mar 3;14:1228. doi: 10.1038/s41467-023-36734-3 (PMC9984388; doi:10.1038/s41467-023-36734-3)
Supplement: Supplementary file 3 — Reporting Summary [file 41467_2023_36734_MOESM3_ESM.pdf]

nature portfolio

Corresponding author(s): Nozomi Ando

Last updated by author(s): Jan 29, 2023

Reporting Summary

Nature Portfolio wishes to improve the reproducibility of the work that we publish. This form provides structure for consistency and transparency in reporting. For further information on Nature Portfolio policies, see our Editorial Policies and the Editorial Policy Checklist.

Statistics

For all statistical analyses, confirm that the following items are present in the figure legend, table legend, main text, or Methods section.

n/a	Confirmed	
☐	☒	The exact sample size (*n*) for each experimental group/condition, given as a discrete number and unit of measurement
☒	☐	A statement on whether measurements were taken from distinct samples or whether the same sample was measured repeatedly
☒	☐	The statistical test(s) used AND whether they are one- or two-sided<br>*Only common tests should be described solely by name; describe more complex techniques in the Methods section.*
☒	☐	A description of all covariates tested
☒	☐	A description of any assumptions or corrections, such as tests of normality and adjustment for multiple comparisons
☐	☒	A full description of the statistical parameters including central tendency (e.g. means) or other basic estimates (e.g. regression coefficient) AND variation (e.g. standard deviation) or associated estimates of uncertainty (e.g. confidence intervals)
☒	☐	For null hypothesis testing, the test statistic (e.g. *F*, *t*, *r*) with confidence intervals, effect sizes, degrees of freedom and *P* value noted<br>*Give P values as exact values whenever suitable.*
☒	☐	For Bayesian analysis, information on the choice of priors and Markov chain Monte Carlo settings
☒	☐	For hierarchical and complex designs, identification of the appropriate level for tests and full reporting of outcomes
☐	☒	Estimates of effect sizes (e.g. Cohen's *d*, Pearson's *r*), indicating how they were calculated

Our web collection on statistics for biologists contains articles on many of the points above.

Software and code

Policy information about availability of computer code

Data collection	Software was specific to the facility (CHESS beamline F1)
Data analysis	XDS 20200131, Aimless 0.7.4, ccp4i2 1.0.2, Phaser 2.8.3, REFMAC 5.8.0267, Coot 0.9.5, MolProbity, MATLAB R2021a, mdx-lib 1.2.0 (https://github.com/ando-lab/mdx-lib), mdx-examples 0.1.1 (https://github.com/ando-lab/mdx-examples)

For manuscripts utilizing custom algorithms or software that are central to the research but not yet described in published literature, software must be made available to editors and reviewers. We strongly encourage code deposition in a community repository (e.g. GitHub). See the Nature Portfolio guidelines for submitting code & software for further information.

Data

Policy information about availability of data

All manuscripts must include a data availability statement. This statement should provide the following information, where applicable:

- Accession codes, unique identifiers, or web links for publicly available datasets
- A description of any restrictions on data availability
- For clinical datasets or third party data, please ensure that the statement adheres to our policy

The raw diffraction data generated in this study have been deposited in the SBGrid Data Bank under accession codes 957 and 958. The atomic coordinates and structure factors have been deposited in the Protein Data Bank (PDB) with accession codes 8dyz and 8dz7. Additionally, this study used diffraction images from

SBGrid Data Bank under accession code 747, processed diffuse maps from the Coherent X-ray Imaging Data Bank (CXIDB) accession code 128, and atomic coordinate files from the PDB with accession codes 6o2h, 193l, 1wtm, and 5l9j.

Human research participants

Policy information about studies involving human research participants and Sex and Gender in Research.

Reporting on sex and gender	N/A
Population characteristics	N/A
Recruitment	N/A
Ethics oversight	N/A

Note that full information on the approval of the study protocol must also be provided in the manuscript.

Field-specific reporting

Please select the one below that is the best fit for your research. If you are not sure, read the appropriate sections before making your selection.

☒ Life sciences ☐ Behavioural & social sciences ☐ Ecological, evolutionary & environmental sciences

For a reference copy of the document with all sections, see nature.com/documents/nr-reporting-summary-flat.pdf

Life sciences study design

All studies must disclose on these points even when the disclosure is negative.

Sample size	We acquired a total of 4000 diffraction images from each crystal form, consisting of a series of 50 degree wedges of 500 images each. Each wedge was collected from different location on one (tetragonal) or two (orthorhombic) large crystals. The number of images (or total exposure time) collected for each crystal was limited by its size and the rate of radiation damage. Due to symmetry of the diffraction pattern, the datasets have a high redundancy: 12-fold for orthorhombic and 24-fold for tetragonal. The high redundancy is leveraged by the data reduction algorithms to correct for experimental artifacts and improve signal-to-noise by averaging.
Data exclusions	Some outlier rejection is performed automatically during scaling and merging of redundant observations. This is necessary because of detector artifacts.
Replication	During data collection sessions, crystals were first screened for diffraction quality before collecting complete datasets. Diffraction images were closely examined for low mosaicity, diffraction resolution, and absence of multiple lattices (such defects can arise if crystals are stressed during handling). The reproducibility of the diffuse pattern for tetragonal lysozyme was confirmed by comparing the reported dataset with one acquired during a prior beam time. This alternative dataset is smaller and of lesser quality, and was therefore not reported.
Randomization	During data processing, the redundant observations are randomly split into two half-datasets for computing the CC* statistic.
Blinding	Data collection and processing were unbiased and performed according to current standards in the field of macromolecular crystallography.

Reporting for specific materials, systems and methods

We require information from authors about some types of materials, experimental systems and methods used in many studies. Here, indicate whether each material, system or method listed is relevant to your study. If you are not sure if a list item applies to your research, read the appropriate section before selecting a response.

Materials & experimental systems

n/a	Involved in the study	
☒	☐	Antibodies
☒	☐	Eukaryotic cell lines
☒	☐	Palaeontology and archaeology
☒	☐	Animals and other organisms
☒	☐	Clinical data
☒	☐	Dual use research of concern

Methods

n/a	Involved in the study	
☒	☐	ChIP-seq
☒	☐	Flow cytometry
☒	☐	MRI-based neuroimaging
